# Clinical characteristics of emergency visits for acute poisoning among children and adolescents: a single-center retrospective study (2021–2025) in China

**DOI:** 10.3389/fped.2026.1939513

**Published:** 2026-10-19

**Authors:** Lin-fei Li, Xian-juan Gou, Jing He, Ling-jun Luo, Li Yang, Mei Chen, Wei-yan Tian

**Affiliations:** Department of Emergency, Affiliated Hospital of Zunyi Medical University, Zunyi, Guizhou, China

**Keywords:** chemical poisoning, drug poisoning, food poisoning, minors, pesticide poisoning

## Abstract

**Objective:**

To investigate the clinical characteristics of acute poisoning in children and adolescents, and to provide a basis for developing targeted prevention and control strategies. This study analyzed the clinical characteristics and temporal trends of acute poisoning cases admitted to the Emergency Department of a tertiary general hospital in Zunyi, Guizhou Province during 2021–2025.

**Methods:**

Data of children and adolescents with acute poisoning presenting to the Emergency Department from January 1, 2021, to December 31, 2025 were collected retrospectively. Descriptive analysis was conducted on age, gender, seasonal distribution and patient discharge disposition.

**Results:**

A total of 1,202 cases of acute poisoning among children and adolescents were enrolled, including 932 valid cases with an effective rate of 77.54%. The annual number of acute poisoning in children and adolescents showed a continuous upward trend from 2021 to 2025, with 40 cases in 2021, 139 cases in 2022, 184 cases in 2023, 240 cases in 2024 and 329 cases in 2025. There were 358 male patients and 574 female patients. Most patients were aged 12–18 years (645 cases, 69.21%), followed by those aged 3–6 years (109 cases, 11.70%), 1–3 years (100 cases, 10.73%), 6–12 years (70 cases, 7.51%), and aged 0–1 year (8 cases, 0.86%). Drug poisoning ranked first (641 cases, 69%), followed by pesticide poisoning (131 cases), chemical poisoning (85 cases) and food poisoning (75 cases). Among patients with drug poisoning, 208 were male and 433 were female. Chemical poisoning, pesticide poisoning and drug poisoning predominantly occurred in the 12–18 age group, while food poisoning was mainly seen in aged 6–12 years. Seasonal distribution analysis showed that the largest number of cases occurred in spring (240 cases, 25.75%), followed by summer (184 cases, 19.74%), autumn (139 cases, 14.91%) and winter (40 cases, 4.29%). Drug poisoning and pesticide poisoning were predominant in spring; drug poisoning and food poisoning were the main types in summer and autumn; drug poisoning and chemical poisoning prevailed in winter. The daily number of drug poisoning was significantly higher than that of the other three poisoning types, with the highest volumes on Mondays and Wednesdays. The afternoon shift received the largest total number of patients (392 cases), especially on Wednesdays (78 cases). The overall intensive care unit (ICU) admission rate for acute poisoning was 7%. The admission rate for pesticide poisoning (84%) was markedly higher than that of other poisoning types. Children with drug poisoning and food poisoning were more likely to receive observation and follow-up.

**Conclusions:**

The number of emergency visits for acute poisoning among minors increased year by year during the study period. Drug poisoning was the leading type, which mainly affected female adolescents aged 12–18 years. Spring was the peak season of onset, and most visits occurred during the afternoon shift. Pesticide poisoning had the highest hospital admission rate. The epidemiological features and clinical outcomes of acute poisoning in local minors are distinct. Clinical and public health interventions should primarily target adolescents and high-risk factors associated with drug poisoning. Targeted health education and early interventions should be implemented according to the risks of severe cases, as well as seasonal and time-based patterns of poisoning, so as to reduce the incidence of acute poisoning and the risk of severe complications.

## 1 Introduction

Acute poisoning is a major global public health threat to the health of children and adolescents and one of the most common critical emergencies in pediatric emergency departments (1). According to data released by the World Health Organization (WHO), 45,000 children and adolescents aged 0–17 years die from acute poisoning worldwide each year. Poisoning ranks the fifth leading cause of unintentional death in children, posing a serious threat to pediatric lives (2, 3).

Acute poisoning refers to a condition characterized by internal environmental disturbance and organ dysfunction after the human body is exposed to toxic substances or excessive doses of drugs within a short period. It has an abrupt onset, severe symptoms, complex toxic mechanisms and rapid disease progression. Delayed treatment may lead to multiple organ injuries, long-term sequelae or even death, imposing substantial socioeconomic and psychological burdens on families and society (4, 5).

Recent study has demonstrated that regional disparities in economic development, living habits and medical resource allocation result in distinct epidemiological characteristics of acute poisoning across different countries and regions (6). A global multicenter study conducted by the International Pediatric Emergency Research Network revealed that acute poisoning accounted for 0.47% of all pediatric emergency visits. A total of 80.6% of poisoning incidents happened at home, oral intake was the primary exposure route (89%), unintentional exposure accounted for 68.5% of cases, and attempted suicide accounted for 13.8% (7). Studies by Berta et al. (8) reported that 72.9% of pediatric acute poisoning cases occurred in children under 5 years old, 85% of poisoning incidents were accidental, and non-pharmaceutical substances were responsible for 59% of cases. Marano et al. (9) found that 74.1% of patients were aged 1–5 years, 98.2% of poisoning cases were accidental, and non-pharmaceutical products caused 66.7% of all poisoning events. Domestic studies have indicated a bimodal age distribution of acute poisoning among Chinese minors. Infants and toddlers aged 1–3 years are mainly poisoned by pesticides and daily chemicals (54.2%), while adolescents aged 12–18 years predominantly take psychotropic and analgesic drugs for self-harm, accounting for more than 80% of cases. Oral ingestion is the main exposure route (96.8%). Pesticide poisoning accounts for up to 51.1% of cases in rural areas, whereas drug poisoning and daily chemical poisoning are more prevalent in urban areas. The proportion of intentional poisoning among adolescents has been rising annually, with the highest incidence in the 16–18 age group (10–12).

Due to immature physical and psychological development, insufficient safety awareness and poor ability to defend against toxins, minors are vulnerable to acute poisoning. Without timely treatment, severe cases may progress to multiple organ dysfunction or failure and result in death (13, 14). As the frontline for receiving and managing acute poisoning patients, emergency departments play a vital role in identifying epidemiological patterns rapidly, which is critical to improving treatment success rates and reducing mortality and sequelae.

This retrospective study analyzed the epidemiological characteristics and spatiotemporal distribution of acute poisoning cases among minors treated in the Emergency Department of a tertiary general hospital in Zunyi, Guizhou Province over five years. The findings aim to clarify the current status of pediatric acute poisoning in this region, provide evidence for optimizing emergency treatment procedures, facilitating rapid diagnosis and targeted treatment, and formulating preventive and intervention strategies. Ultimately, this study helps reduce the disease burden of acute poisoning and protect the health of minors.

## 2 Methods

### 2.1 Study design

This was a retrospective cross-sectional study. Medical records of patients presenting to the Emergency Department between January 1, 2021, and December 31, 2025, were extracted from the Hospital Information System (HIS) using the keyword “poisoning”. Inclusion criteria: (1) Patients younger than 18 years old; (2) Complete medical records. Exclusion criteria: Patients with metabolic acidosis, metabolic alkalosis, respiratory acidosis, respiratory alkalosis, diabetic ketoacidosis, toxic encephalopathy, and poisoning caused by animal stings or bites (including insect and snake bites).

### 2.2 Data collection

Two researchers independently extracted and cross-checked all data in strict accordance with data management standards to ensure reliability. Cases with missing key variables were excluded from statistical analysis. The interquartile range method was used to screen outliers. All abnormal records were verified against original medical files: erroneous entries were corrected, authentic extreme values were retained, and untraceable records were excluded.

### 2.3 Definitions

Poisoning classification: Referring to Complete Book of Acute Poisoning (2nd Edition) and annual reports of the National Poison Data System (NPDS) of the United States, poisoning substances were divided into four categories: chemicals, pesticides, drugs and foods (15, 16). According to emergency medical records, patient discharge disposition was classified as admission to ICU, admission to general ward, follow-up, observation, and discharge against medical advice. Work shifts: Based on the local scheduling rules of the emergency department, shifts were divided into the morning shift (8:00 a.m. – 4:00 p.m.), afternoon shift (4:00 p.m. – 11:00 p.m.) and night shift (11:00 p.m. – 8:00 a.m.). Seasons: Seasons were divided according to Chinese meteorological standards: Spring (March, April, May); Summer (June, July, August); Autumn (September, October, November); Winter (December, January, February).

### 2.4 Ethical approval

The study protocol was reviewed and approved by the Ethics Committee of The Affiliated Hospital of Zunyi Medical University (No. KLL-2024-581). The study was performed in accordance with the Declaration of Helsinki.

### 2.5 Statistical analysis

Descriptive statistical methods were applied for data analysis. Microsoft Excel was used for data summary, case counting, and calculation of frequencies and constituent ratios. Origin software was adopted to draw stacked bar charts, Pareto charts and heat maps to visualize the distribution of cases in terms of gender, age, poisoning types, year, season, weekday and work shift, as well as the discharge disposition of patients with different types of poisoning.

## 3 Results

### 3.1 Frequency and cumulative percentage of different poisoning types

A total of 1,202 cases of acute poisoning were collected, and 932 valid cases were included, with an effective rate of 77.54%. Among the excluded 270 cases, 65 had metabolic or respiratory acid-base disorders; 152 had missing core variables in medical records and the data could not be traced; 32 were diagnosed with diabetic ketoacidosis; 12 had toxic encephalopathy; and 9 were poisoning caused by animal stings or bites. As shown in Figure 1, drug poisoning was the most common type, involving 641 cases and accounting for 69% of all valid cases. Pesticide poisoning ranked second (131 cases), followed by chemical poisoning (85 cases) and food poisoning (75 cases). In accordance with the Pareto principle (80/20 rule), the cumulative proportion of drug, pesticide and chemical poisoning reached 92%, exceeding the 80% threshold, indicating that these three categories of toxic substances were the major high-risk factors for acute poisoning among local minors.

**Figure 1:**
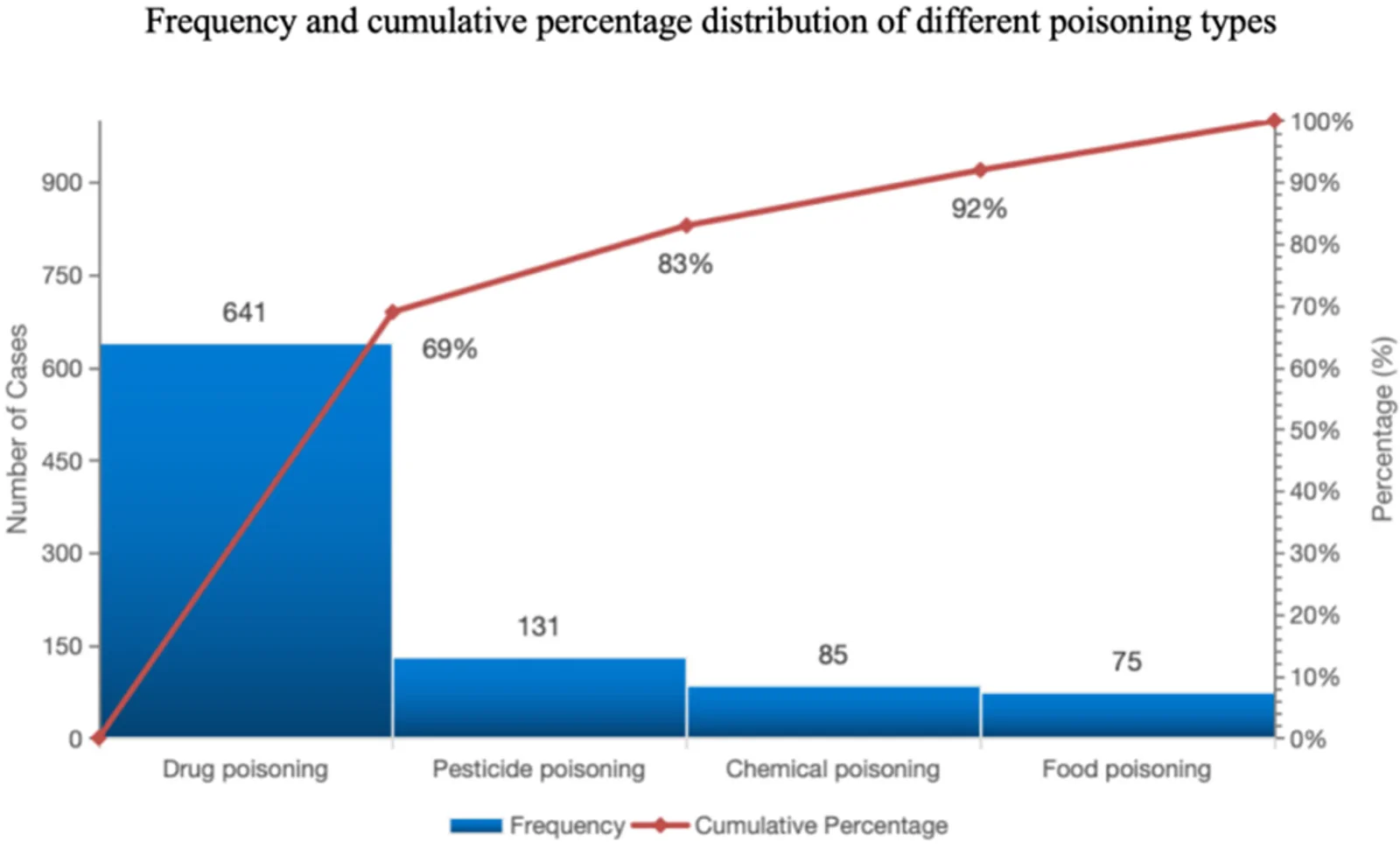
Pareto chart of case frequency and cumulative percentage of different acute poisoning types among minors. Blue bars represent the number of cases for each poisoning type; the red line represents the corresponding cumulative percentage.

### 3.2 Gender and age distribution of acute poisoning cases

As shown in Figure 2, among all patients, 358 were male (38.41%) and 574 were female (61.59%). In terms of poisoning types by gender, drug poisoning was the most prevalent in both genders, with 208 male cases and 433 female cases. The numbers of patients with pesticide, chemical and food poisoning were relatively small, with slightly more male patients or a similar gender ratio. The 12–18 age group had the largest number of cases (645 cases, 69.21%), followed by the 3–6 age group (109 cases, 11.70%), 1–3 age group (100 cases, 10.73%), 6–12 age group (70 cases, 7.51%), and the 0–1 infant group (8 cases, 0.86%). In terms of age distribution across poisoning types, drug poisoning occurred across all age groups: 6 cases in the 0–1 age group, 77 cases in the 1–3 age group, 60 cases in the 3–6 age group and 482 cases in the 12–18 age group. Food poisoning was predominantly seen in children aged 6–12 years.

**Figure 2:**
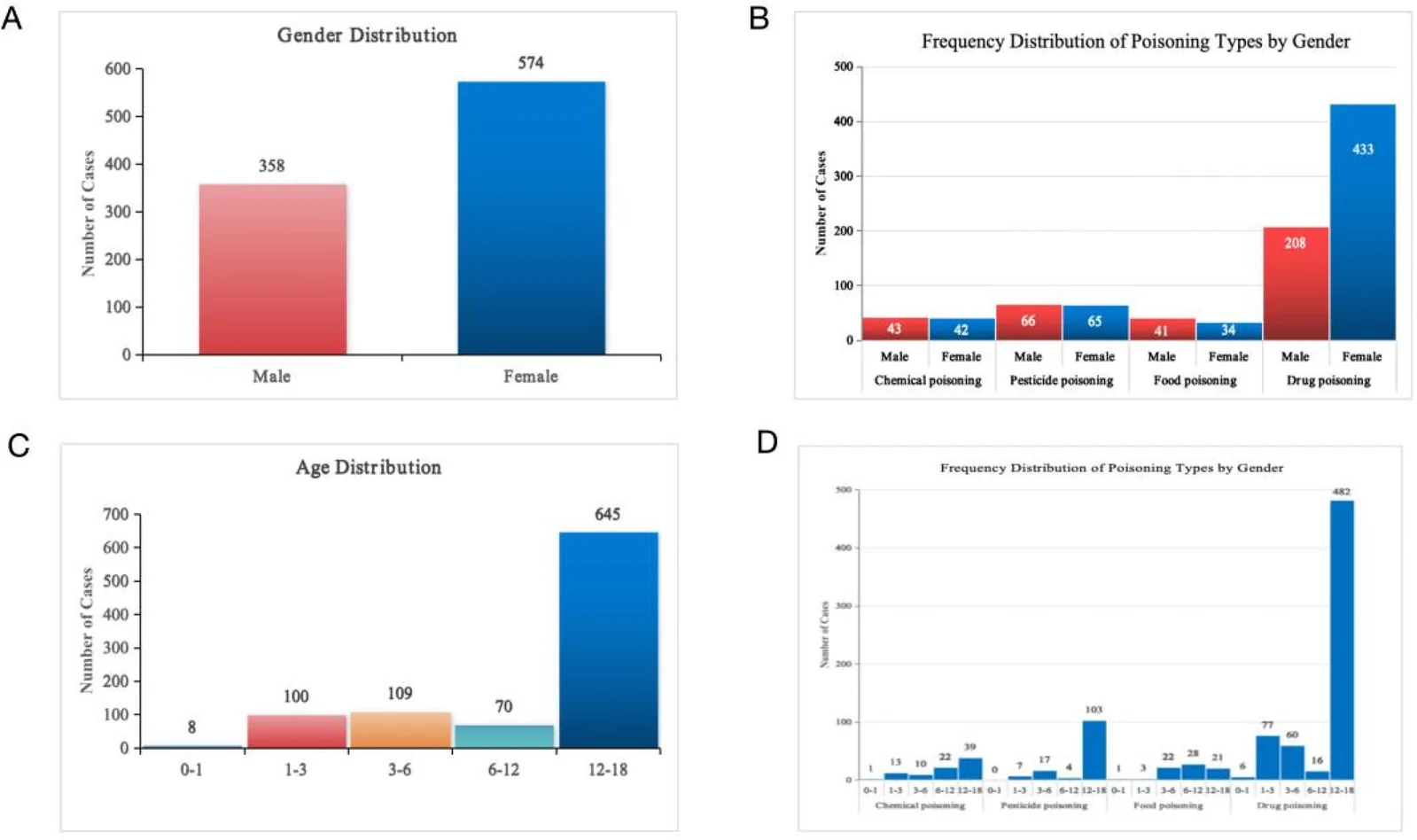
Gender and age distribution of acute poisoning cases. **(A)** showing genderbased case distribution, with 358 male cases and 574 female cases. **(B)** presenting the case frequency of four poisoning types stratified by gender (chemical, pesticide, food, and drug poisoning). **(C)** illustrating case distribution across five age subgroups: 01year, 13years, 36years, 612years, and 1218years. **(D)** displaying the frequency of different poisoning types among each age group.

### 3.3 Spatiotemporal distribution of acute poisoning cases among minors

As shown in Figure 3, the annual number of acute poisoning cases increased steadily from 2021 to 2025: 40 cases in 2021, 139 cases in 2022, 184 cases in 2023, 240 cases in 2024 and 329 cases in 2025. Drug poisoning remained the predominant type across all years.

**Figure 3:**
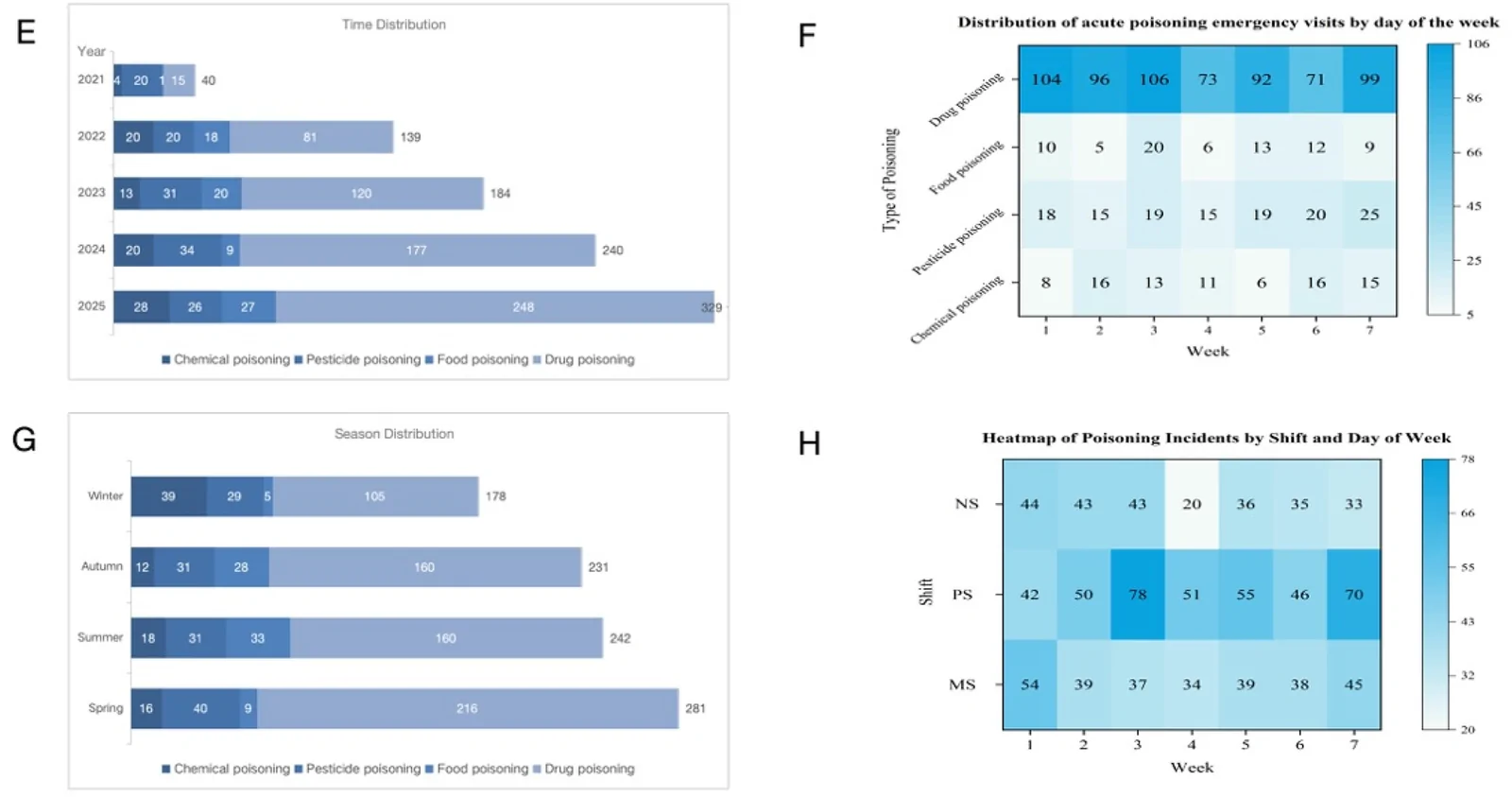
Spatio-temporal distribution of emergency visits for acute poisoning. **(E)** showing the annual based case composition of four poisoning types from 2021 to 2025. **(F)** illustrating the distribution of emergency visits for different poisoning types across days of the week. **(G)** presenting seasonal case composition of four poisoning types. **(H)** displaying the number of poisoning incidents stratified by work shift and day of the week. MS, morning shift; PS, postmeridian shift (Afternoon Shift); NS, night shift.

For the seasonal distribution of all acute-poisoning cases, spring had the largest number of cases (281 cases), followed by summer (242 cases), autumn (231 cases), and winter (178 cases). Drug poisoning and pesticide poisoning were predominant in spring; drug poisoning and food poisoning were the main types in summer and autumn; drug poisoning and chemical poisoning were most common in winter.

The daily number of visits for drug poisoning was significantly higher than that of the other three poisoning types, with peak volumes on Mondays (104 cases) and Wednesdays (106 cases).

Regarding work-shift distribution, 254 cases presented during the morning shift, 392 cases during the afternoon shift, and 286 cases during the night shift. The Wednesday afternoon shift recorded the highest case volume (78 cases), followed by the Sunday afternoon shift (70 cases).

### 3.4 Discharge disposition of patients with different types of acute poisoning

As shown in Figure 4, patients with pesticide poisoning had the highest rate of admission to general wards (73%) and ICU admission (13%). Food poisoning had the highest rates of follow-up care (37%). Drug poisoning had the highest rates of discharge against medical advice (20%) and emergency observation (24%).

**Figure 4:**
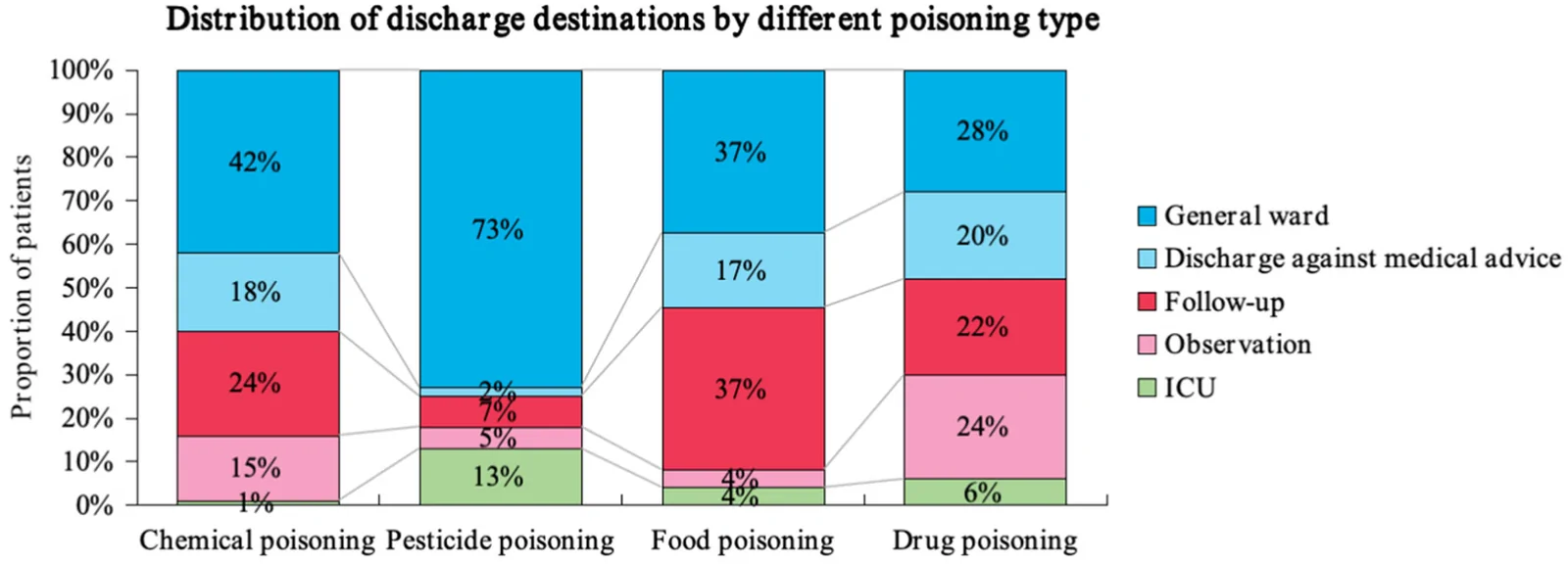
Discharge disposition of patients with different types of acute poisoning.

## 4 Discussion

This study investigated the epidemiological characteristics of acute poisoning among minors in the local region. The results revealed a continuous increase in annual emergency visits for acute poisoning. Drug poisoning was the leading type, with a higher incidence in female patients and adolescents aged 12–18 years. The peak visits occurred in spring, during the afternoon shift and on Wednesdays.

The rising trend of acute poisoning visits from 2021 to 2025 was consistent with findings of previous study (5), which was closely associated with adjustments to COVID-19 prevention and control policies. During the pandemic period from 2021 to 2022, nationwide public health interventions including movement restrictions and suspension of kindergartens and schools reduced social mobility and limited minors' access to toxic substances. Meanwhile, some patients with mild symptoms delayed medical treatment due to limited medical resources, leading to fewer poisoning visits (17). After the pandemic, optimized control policies restored normal social activities and improved medical accessibility, releasing previously suppressed medical demands. In addition, increased psychological stress after the resumption of regular life further contributed to the rebound and growth of acute poisoning cases (18).

Drug poisoning was the primary type of acute poisoning among local minors (641 cases, 69.21%), with a higher incidence in females and adolescents aged 12–18 years, which was in line with previous domestic and international studies (5, 19–22). This disproportionate predominance of drug poisoning was mainly attributed to the growing prevalence of mental health problems among adolescents. Academic pressure and family conflicts are common triggers for intentional drug overdose (23). Studies have confirmed that females have a higher incidence of mental disorders, including depression, emotional crises during adolescence, and hormonal fluctuations (24). Mental health intervention services for adolescents are insufficient in this region. Regular psychological screening and emotional counseling should be prioritized for female adolescents in campus settings, and relevant training should be provided for parents and medical staff to strengthen poisoning prevention. A three-dimensional protection network covering schools, families and hospitals should be established for the prevention and management of adolescent poisoning. Researcher has also suggested stricter prescription management of psychotropic drugs and routine mental health screening for adolescents. Parents and educators should pay close attention to the mental status of teenagers and provide timely psychological support and education (5).

Acute poisoning occurred all year round, with the highest incidence in spring and summer, mainly involving drugs and pesticides, which was consistent with global research findings (1, 25–27). The increased use of pesticides during the spring farming season raises the risk of accidental exposure and ingestion among minors. In addition, one study has identified academic pressure as a risk factor for intentional self-harm among adolescents. Elevated academic stress experienced by adolescents following the start of the new spring semester may be associated with a rise in poisoning cases (28). The number of daily visits for drug poisoning was consistently higher than other poisoning types, with weekly peaks on Mondays and Wednesdays. This trend is related to academic stress cycles: students may experience anxiety at the start of school on Mondays and fatigue in the middle of the week on Wednesdays, triggering emotion-related drug poisoning incidents. In addition, reduced adult supervision on workdays also increases the risk of accidental ingestion among young children (15). During this period, children have more outdoor activities, while caregivers tend to be distracted; emotional conflicts among adolescents after school also contribute to more poisoning events (29). Fewer cases occurred during the night shift, as minors are mostly asleep and have limited access to toxic substances. Differentiated prevention and control strategies should be implemented according to seasonal features: prioritize pesticide safety management and adolescent mental health screening in spring; strengthen food safety and household management of drugs and chemicals in summer and autumn; and reinforce home safety education in winter. Emergency department staffing should be optimized based on time-based visit peaks: allocate more medical resources during the afternoon shift while maintaining basic staffing for the night shift, so as to improve both treatment efficiency and resource utilization.

According to the 2024 annual report of the United States National Poison Data System (NPDS), the ICU admission rate for pediatric acute poisoning ranges from 3% to 8% (30). A single-center study in Italy reported a pediatric intensive care unit (PICU) admission rate of 5% (27). The overall ICU admission rate of 6% in this study was comparable with international data, indicating that most cases of pediatric acute poisoning were mild, and only a small proportion of patients developed critical conditions requiring advanced life support in the ICU (31–33). Pesticides have strong cytotoxicity and can rapidly cause respiratory and circulatory failure, neurological damage and multiple organ dysfunction, with a high risk of delayed complications. Pesticide poisoning is an independent risk factor for poor clinical outcomes in children (34). In this study, 73% of children with pesticide poisoning required hospital admission and 13% were admitted to the ICU, suggesting that pesticide poisoning is generally severe and necessitates in-hospital continuous monitoring, detoxification, and symptomatic supportive care. In this study, drug poisoning was the most common type of poisoning. Since most patients received observation or were discharged against medical advice, the observed hospital admission rate was therefore relatively low. However, this also reflects that some patients and their guardians lack awareness of delayed adverse reactions and have poor treatment compliance. The varied discharge dispositions across different poisoning types directly reflect differences in disease severity, treatment requirements and patient compliance. The results can provide references for optimizing clinical treatment procedures, strengthening health education and improving follow-up systems.

Poisoning prevention education can effectively improve people's cognition of medication safety and reduce risky behaviors (35). Joint efforts from families and society are essential for poisoning prevention. At the family level, drugs, chemicals and pesticides should be stored in locked and elevated cabinets with original packaging retained; expired articles should be cleaned up regularly. At the social level, age-stratified safety education should be carried out to help minors recognize hazard signs, combined with mental health intervention and crisis counseling (36). Schools should improve safety protection measures and deliver regular safety education courses tailored to children of different ages. Medical institutions should cooperate to launch a 24-hour national poison consultation and emergency hotline to provide real-time guidance for accidental ingestion and timely referral and set up dedicated emergency green channels for poisoning patients (37).

## 5 Conclusions

Acute poisoning among local minors presents distinct epidemiological characteristics, including specific high-risk toxic substances, vulnerable populations, obvious seasonal patterns and unique clinical outcomes. Clinical managers should implement stratified diagnosis, treatment and management strategies based on age groups and poisoning types. Priority should be given to the source control of drug, pesticide and chemical poisoning among adolescents. Targeted health education and early interventions are recommended. Emergency staffing should be strengthened in peak seasons and time periods to reduce the overall incidence of acute poisoning and the risk of severe illness.

## 6 Limitations

This study has several limitations. First, all data were collected from a single hospital, which may lead to selection bias. Multicenter and large-sample studies are needed to verify the present findings in the future. Second, this is a retrospective study. All data in this study were extracted from the hospital's electronic medical record system. Information regarding poisoning routes, inducements, and toxic doses was unavailable; only broad categories such as food poisoning and drug poisoning were recorded, without specific drug names. This finding also reminds clinical staff to standardize medical documentation for poisoning cases. Third, this study only conducted descriptive analysis of epidemiological features. No risk prediction model was established, and no long-term follow-up was performed to quantify independent influencing factors and long-term outcomes of different poisoning types. Further multicenter prospective studies combining social environment, family factors and psychological characteristics are required to improve the risk early warning and precise prevention system for acute poisoning among minors. In addition, changes in COVID-19 public health restrictions may partly explain the observed upward trend in pediatric acute poisoning visits. As this is a retrospective observational study, however, no causal inference can be established, and this temporal trend should be interpreted with caution.

## Data Availability

The raw data supporting the conclusions of this article will be made available by the authors, without undue reservation.
